# Detection and Quantification of the Oomycete *Saprolegnia parasitica* in Aquaculture Environments

**DOI:** 10.3390/microorganisms10112186

**Published:** 2022-11-03

**Authors:** Tiina Korkea-aho, Tom Wiklund, Christine Engblom, Anssi Vainikka, Satu Viljamaa-Dirks

**Affiliations:** 1 Animal Health Diagnostic Unit, Finnish Food Authority, 70210 Kuopio, Finland; 2 Laboratory of Aquatic Pathobiology, Environmental and Marine Biology, Åbo Akademi University, 20520 Turku, Finland; 3 Department of Environmental and Biological Sciences, University of Eastern Finland, 80101 Joensuu, Finland

**Keywords:** fish pathogen, oomycete, *Saprolegnia parasitica*, qPCR assay, eDNA detection

## Abstract

*Saprolegnia parasitica* induces heavy mortality in aquaculture. The detection of *S. parasitica* is often time consuming and uncertain, making it difficult to manage the disease. We validated a previously published real-time quantitative PCR (qPCR) assay to confirm the presence of *S. parasitica* in fish and in water using environmental DNA (eDNA) quantification. Analytical sensitivity and specificity of the assay was assessed in silico, in vitro and the qPCR assay was compared with microbiological cultivation methods to detect and quantify *S. parasitica* in water samples from a controlled fish exposure experiment and from fish farms. Furthermore, we compared the use of an agar cultivation method and the qPCR assay to detect *S. parasitica* directly from mucus samples taken from the fish surface. The analytical sensitivity and specificity of the qPCR assay were high. The qPCR assay detected 100% of *S. parasitica*-positive water samples. In a field study, the qPCR assay and a microwell plate (MWP) enumeration method correlated significantly. Furthermore, the qPCR assay could be used to confirm the presence of *S. parasitica* in skin mucus. Thus, the qPCR assay could complement diagnostic methods in specifically detecting saprolegniosis in fish and used as a surveillance method for *S. parasitica* pathogen in aquaculture environments.

## 1. Introduction

Oomycetes of the genus *Saprolegnia* cause the disease saprolegniosis in fish, with considerable losses, especially in freshwater aquaculture. *Saprolegnia parasitica* has frequently been isolated from diseased salmonids in Finland [1] and elsewhere [2,3,4,5]. It is considered as the most pathogenic oomycete for juvenile and adult salmonids, while other *Saprolegnia* species are more devastating for fish eggs and fry [6,7,8]. The infection can be seen on fish as greyish and whitish hyphae growing on the skin and gills. The disease induces heavy mortality in fish when hyphae penetrate through fish tissues, causing impaired osmoregulation or respiratory failure. Saprolegniosis is a major problem for fish welfare and the economic sustainability of fish farming, as there is no known effective treatment for the disease in fish [6,9]. Thus, accurate detection and diagnosis of *S. parasitica* in fish and the aquaculture environment are crucial for the development of any effective control measures to prevent disease outbreaks on fish farms.

The recognition of oomycete species still mainly relies on isolation and morphological examination and/or internal transcribed spacer (ITS) sequencing of the isolates [4,5]. These methods are laborious and time consuming, as isolation requires cultivation of the microorganisms. Furthermore, morphological identification is based on ambiguous morphological characters of *Saprolegnia* spp. (e.g., oogonia production), and recognition may not always be repeatable under laboratory conditions [7,10]. Moreover, ITS sequences of *Saprolegnia* spp. in the public GenBank are not complete, and *Saprolegnia* ITS sequences have been designated erroneous species names [7], which makes *Saprolegnia* species identification based on ITS sequence comparison difficult.

A real-time quantitative PCR (qPCR) assay would be an accurate and rapid method to detect and quantify the species *Saprolegnia parasitica* from fish and aquaculture environments. Rocchi et al. [11] established a qPCR assay that targets the ITS region of *S. parasitica*. They applied it to quantify *S. parasitica* in water from a river that had a history of massive fish mortalities caused by *S. parasitica,* as well as municipal water taken from the river. Methods for the detection of environmental DNA (eDNA) are emerging in species monitoring, especially in aquatic environments. eDNA methods are also used to detect various aquatic pathogens [12,13,14]. The World Organization for Animal Health (WOAH) recognizes that eDNA methods have several advantages over other methods in the early detection of pathogens and in monitoring [15]. However, eDNA methods require careful validation, as they must be specific to the species targeted and sensitive enough to detect target DNA in low quantities. Thus, sufficient validation of an eDNA method requires in silico, in vitro and field tests with the preliminary protocol [16]. A qPCR assay, which could be used for accurate species identification in fish disease diagnostics, as well as for recognizing the target DNA in environmental samples, could be very useful in understanding the occurrence of saprolegniosis disease outbreaks and the interactions between the host, pathogen and environment.

In this study, our aim was to test the validity of the published qPCR assay [11] for *S. parasitica* recognition in fish and in water samples from the aquaculture environment by (1) comparing the sequences of the *S. parasitica* qPCR primers and probe with the ITS sequences of a large number of Finnish *S. parasitica* isolates, as well as GenBank isolates in silico, (2) testing the sensitivity and specificity of the qPCR assay in vitro and (3) comparing eDNA assay with several microbiological methods for the detection and quantification of *S. parasitica* in water samples collected from fish farms.

## 2. Materials and Methods

Several methods were used in this research to validate qPCR for the detection and quantification of *S. parasitica*. For the in silico and in vivo analysis, oomycete information and pure cultures were used from the oomycete culture collection of the Finnish Food Authority. To compare qPCR and microbiological methods, water samples were analysed from an exposure experiment in which fish were exposed to *S. parasitica* added to the tank water. Water samples were collected from the fish tanks and analysed several times during the 16 days of the exposure experiment. Finally, to validate the use of the eDNA qPCR method in the aquaculture environment, samples for analysis were collected from four fish farms and from one lake, and qPCR results were compared with those of the microwell plate (MWP) enumeration method. Furthermore, fish from the exposure experiment and the fish farms were analysed for the presence of *S. parasitica* using qPCR and microbiological cultivation.

### 2.1. Oomycete Collection, Cultivation and DNA Extraction

To obtain representative oomycete samples, isolates of Saprolegniaceae were collected from several infected salmonids in different geographical areas of Finland. Pure cultures of the isolates were produced and subsequently identified based on ITS sequences [1] and multilocus sequence typing (MLST) (unpublished data). For comparison, related *Aphanomyces* spp. cultures from crayfish and pikeperch were selected from the oomycete culture collection of the Finnish Food Authority (Table 1). The pure cultured oomycete strains were stored at +4 °C on peptone glucose agar (PG-1) [17] in tubes with sterile paraffine on top. For qPCR specificity analysis, a piece of mycelium was re-cultivated on PG-1 agar at 15 °C for 3-7 days. The grown mycelium (ca. 2 mm^3^ in size) was excised aseptically from the agar plate and placed in sterile 2-mL tubes with beads (NucleoSpin Bead Tubes, Macherey-Nagel, Düren, Germany) for DNA isolation. For qPCR sensitivity analysis, mycelium of *S. parasitica* strain VH28 was grown in PG-1 broth at 20 °C for three days. The mycelium was rinsed with sterile MilliQ water, and 65 mg of mycelium was cut and placed in 2-mL tubes with beads and stored at -20 °C before DNA isolation.

DNA from oomycete isolates, fish and water samples was isolated using the DNeasy Blood and Tissue kit and a QIAcube DNA extraction Robot (Qiagen, Hilden, Germany) according to the spin-column protocol for purification of total DNA from animal tissues. At the beginning of extraction, sterile 1×PBS was added to the samples, and they were homogenized in 2-mL bead tubes with a MagNA Lyser instrument (Roche Diagnostic GmbH, Germany), which was followed by cell lysis according to the protocol. In each extraction series, every 12th extraction tube contained sterile 1× PBS without a sample, and these were included in qPCR plates as samples to control for potential contamination in the DNA extraction. DNA was eluted in 100-µL volumes and stored at −20 °C.

### 2.2. S. parasitica qPCR Assay Validation

The qPCR assay published by Rocchi et al. [11] for *Saprolegnia parasitica* was first validated in silico by comparing the complementarity of the sequences of the primers and probes for ITS region sequences of the *S. parasitica* isolates from Finnish fish farms [1] and against the National Centre for Biotechnology Information (NCBI, https://www.ncbi.nlm.nih.gov/) (last accessed on 12 September 2022) GenBank database using the Basic Local Alignment Search Tool for nucleotides (BLASTn). Second, the specificity of the qPCR assay was analysed in vitro using the cultured isolates of oomycetes, including *S. parasitica* strains from various salmonids (see above) and closely related oomycete strains (Table 1). For sensitivity analysis of the qPCR, different concentrations of *S. parasitica* VH28 DNA were used. The elution concentration of *S. parasitica* VH28 DNA was determined with a Nanodrop ND-1000 spectrophotometer and a 10-fold dilution using PCR-grade UltraPure™ water (Invitrogen, Paisley, UK) for qPCR standard curve analysis.

The analytical specificity and sensitivity and the limit of detection (LOD) of the qPCR assay were determined in vitro. The qPCR reactions were run with the QuantStudio 5 Real-Time PCR System (Applied Biosystems, Thermo Fisher Scientific, Life Technologies, Carlsbad, CA, USA) with primers and probe amplifying the *Saprolegnia parasitica* ITS2—28S region with primer-F (5′-AGAGCAAATCGCGGTAGTTT-3′), primer-R (5′-AGAAATGCACCAGCATACCA-3′) and probe-R (5′-FAM-TGCCTTGTACTTTGACAACAGACTCGC-BHQ2-3′) [11]. For pure culture isolates, the final volume of the PCR reaction was 22 µL, with 11 µL of SensiFAST Probe No-ROX mix (2×) (Meridian Bioscience, Cincinnati, OH, USA), 2 µL of DNA template, 400 nM of primers, 200 nM of probes and PCR-grade water. For water and fish samples, the final volume of the PCR reaction was 25 µL with 12.5 µL of 2× SensiFAST Probe No-ROX mix(2×), 5 µL of DNA template and the same content of primers and probes as the previous PCR assay. The PCR protocol was 5 min at 95 °C followed by 40 cycles: 10 s at 95 °C and 30 s at 60 °C. To detect inhibition, DNA samples from the isolates were always also run as a 1:10 dilution, and the pUC 18 plasmid of about 5000 copies was used as an internal amplification control (IAC) in the PCR reactions for fish and water samples. For IAC pUC amplification primers and probe were used [18]. The analytical sensitivity and LOD were determined for the PCR assay with and without an IAC. Inhibition was only detected in some water samples; these samples were re-run in PCR as a 10-fold dilution.

In addition, with the blank DNA extraction control template, every 13th well in the PCR run contained only PCR-grade water as a no-template control (NTC) sample. Furthermore, *Aphanomyces astaci* strain Da was included in each PCR run as a negative control sample. Each analytical PCR assay was performed in six replicates. When analysing the samples from the exposure experiment (see below) and from fish farms, all the templates were always pipetted as duplicates or triplicates in the PCR plate. All the analyses were performed in a laboratory complying with the ISO/IEC 17025 standard.

### 2.3. Comparison of the qPCR Assay and Microbiological Methods to Detect S. parasitica in Water and Fish Samples Using an Exposure Experiment

An exposure experiment involving brown trout (*Salmo trutta*) using isolate VH28 of *S. parasitica* (Table 1) was conducted under laboratory conditions to validate the ability of the test to reveal developing disease in fish and the tank environment. The brown trout (mean weight ± standard deviation (SD) = 13.4 ± 2.3 g, mean length ± SD = 114 ± 5 mm) were acquired from a commercial hatchery and were acclimated in a fish research facility for 10 days. The water temperature in all tanks was maintained at an average (±SD) of 14.3 ± 1.2 °C during the experiment. The fish were held in tanks with a water volume of 120 L, constant water flow (5 L/min) of dechlorinated municipal water and a 12 h photoperiod. On arrival, the fish were bathed for 20 min in formaldehyde (35% solution) diluted 1:5000 in aerated standing water to eliminate possible oomycete spores on fish or transport water. They were fed daily with commercial pellet (Hercules, Raisioaqua) at 1% of their body weight. Three days after formalin bathing, 10 fish were randomly selected and killed with an overdose of benzocaine (MP Biomedicals, LLC, Illkirch, France). The health status of all ten fish was checked by pathological and microbiological analysis and no disease symptoms or pathogens were found in any of the fish.

After acclimation, the fish were randomly allocated to six tanks (1–6) similar to the holding tanks, with 17 fish in each tank. Three treatment groups were established with 34 fish per treatment in two tanks: (1) *S. parasitica* (tanks 1 and 2), (2) *S. parasitica* + injection (tanks 3 and 4) and (3) control (tanks 5 and 6). Prior to the allocation to new tanks, the fish were anaesthetized with 0.05 g/L benzocaine in aerated water. For *S. parasitica* + injection treatment in tanks 3 and 4, fish were injected intramuscularly (i.m.) with 50 µL of sterile 1/4 diluted nutrient broth (0.075% beef extract, 0.125% peptone, pH 6.8) into the epaxial muscle to mimic handling stress associated with vaccination. *S. parasitica* of the strain VH28 was grown in PG-1 broth and sporulation was induced as described by Dieguez-Uribeondo et al. [19]. The spore concentration was adjusted to 4.3 × 10^6^ spores/L in MilliQ water and confirmed by manual counting using a haematocytometer. After five days from the beginning of the experiment, the water volume in all the tanks was reduced to 34 L, the water flow was stopped, and the tank water was aerated. *S. parasitica* VH28 was added in quantity of 236 mL in the *Saprolegnia* and *Saprolegnia* + injection treatment tanks 1, 2, 3 and 4, to achieve final concentration of 3 × 10^4^ *S. parasitica* spores/L. The same volume of pure MilliQ water was added to the control treatment tanks 5 and 6. After 20 h, water samples were taken from the tanks (day 1) and the whole water volume was replaced with fresh water. Thereafter, the water volume in the tanks was maintained at 200 L and the water was changed daily until the end of the experiment, i.e., 16 days. On day 16, all the fish were killed with an overdose of benzocaine. The fish were weighed, measured for length and clinical signs were recorded. Samples for microbiological analysis were taken with a sterile loop from the spleen and kidney and streaked on 1/4 diluted nutrient agar (1/4 NA; 0.075% beef extract, 0.125% peptone, pH 6.8). Mucus of the fish was scraped from one side of the body above the horizontal septum with a sterile scalpel and added to a sterile tube containing 0.5 g of 0.5 mm ceramic beads (OMNI International Inc. Kennesaw, GA, USA) and 450 µL of 1x PBS. The tube was vortexed briefly and 100 µL was plated on replicate PG-1 agar plates with antibiotics (ampicillin and oxolinic acid 10 µg/mL) [19], while the rest of the sample in the tube was stored at −20 °C for DNA extraction. The agar plates were incubated at 15 °C for 7 days and inspected every second day for bacterial or hyphal growth.

During the *S. parasitica* exposure experiment, water samples were taken from each tank on days 1, 4, 7 and 15 after the initial exposure challenge. Water samples were taken approximately 10 cm below water surface directly into two 15 mL falcon tubes (2 × 15 mL per sampling) and placed on ice. The 15 mL water samples were filtered through 0.45-µm Supor^®^ Membrane Disc Filters (Pall Life Sciences, Ann Arbor, MI, USA) using a vacuum pump. The membrane filters were aseptically rolled and cut into smaller pieces that were placed in 2 mL tubes with beads and stored at −20 °C.

To compare the qPCR assay against more traditional microbiological methods, water samples from the exposure experiment were also investigated for *Saprolegnia* spp. with microbiological methods. To isolate *Saprolegnia* spp., 100 µL of water sample was added onto two replicate PG-1 agar plates containing antibiotics (ampicillin and oxolinic acid, 10 µg/mL), and duplicate of 1 mL of water were additionally added to two wells of a sterile 4-well dish (Nunc, Thermo Scientific, Roskilde, Denmark), containing 500 µL PG-1 broth with antibiotics (ampicillin and oxolinic acid, 10 µg/mL). Furthermore, three autoclaved hemp seeds per well were incubated in replicate wells with 1 mL of sampled water, as hemp seeds are commonly used in *Saprolegnia* spp. bating from water [11,20]. All water samples were incubated at 15 °C for 48 h. If hyphal growth was detected, the isolate was confirmed at the genus level as *Saprolegnia* by inducing sporulation, as described by Dieguez-Uribeondo et al. [19] with minor modifications. Briefly, a small piece of hyphae was washed three times in a drop of sterile MilliQ water. The hyphae were subsequently incubated at +20 °C overnight in sterile MilliQ water and examined microscopically (×100 magnification) for maturing zoosporangium and zoospore discharge, as these are typical of the hyphal growth and characteristics of *Saprolegnia* [20,21].

The challenge experiment was conducted according to Directive 2013/63/EU on the protection of animals used for scientific purposes and the Act and Governmental Decree on the Protection of Animals Used for Scientific or Educational Purposes (497/2013 and 564/2013, respectively). The challenge experiment had been reviewed and approved by the National Animal Experiment Board at the Regional State Administrative Agency for Southern Finland with permission ESAVI/16637/2019.

### 2.4. Quantification of S. parasitica from Aquaculture Environments

Water samples were collected from four fish farms, whose identities are not revealed for confidentiality. Three tanks containing fish on each farm were sampled once (i.e., three water samples per farm). One natural water sample was collected for comparison from the Savilahti shore of Lake Kallavesi. Fish farms 1, 3 and 4 were flow-through systems and fish farm 2 a recirculating aquaculture system (RAS). Water samples were taken in 500 mL sterile high-density polyethylene water sampling bottles containing 20 mg/L sodium thiosulphate (Avantor, VWR International, Leuven, Belgium) from near the outflow of the fish tanks. The samples were kept cool, and subsamples were filtered within 24 h in a laboratory, as described for the water samples from the exposure experiment. For each water sample, three 15 mL samples were filtered using 0.45-µm Supor^®^ Membrane Disc Filters. The filters were kept at −20 °C until DNA was extracted, and qPCR was performed as described earlier. For the standard curve analysis, a standard dilution series of *S. parasitica* VH28 down to 10^−6^-fold with a known DNA concentration was run in each qPCR and used as the target amount of *S. parasitica* DNA in the quantification of unknown water samples using a regression equation. The mean and standard deviation were calculated for the amount of *S. parasitica* DNA (ng/L) in tank water from triplicate filters. In addition, *Saprolegnia* spore counts from the same water samples were estimated using MWP method [20]. Briefly, 100 µL of PG-1 broth containing antibiotics (ampicillin and oxolinic acid, 10 µg/mL) was added to each well of a 96-microwell plate (Nunc, Thermo Scientific, Roskilde, Denmark). The water sampling bottle was then shaken vigorously and 100 µL of water was added to each well. Three 96-microwell plates were prepared for each water sample. With each sampling, one control 96-microwell plate was included, in which 100 µL PG-1 containing antibiotics and 100 µL of sterile MilliQ water was used as a water sample. The microwell plates were incubated at +20 °C and checked after 24 h and 48 h for hyphal growth. Growing hyphae from at least three wells from each plate were further sporulated and confirmed as *Saprolegnia* as described before. Hyphal growth was assessed with a stereomicroscope and each well displaying typical growth of *Saprolegnia* was considered as one spore in 9.6 mL of water sample (100 µL water sample per well × 96 wells) [20]. A multiplication factor of 104.16667 (1000/9.6) was used to estimate the spore number in one litre of water. The mean and standard deviation were calculated for the spore number per litre of the tanks water from triplicate microwell plates.

In addition to the water samples, five fish were sampled from each tank and killed by a blow to the head, after which they were transported to the laboratory on ice and investigated pathologically and microbiologically. Mucus samples were scraped from the fish as described previously and the mucus samples were tested for the presence of *S. parasitica* by qPCR. Furthermore, if *Saprolegnia* growth was seen macroscopically on fish, a small piece of hyphae growing on the skin or gills was excised from the fish, rinsed in 70% ethanol and MilliQ water, placed on PG-1 agar containing antibiotics and incubated at +15 °C for 7 days. The plates were checked for hyphal growth every second day. Hyphae were re-cultivated on PG-1 agar containing antibiotics if needed until no bacterial growth was seen on the plate. A piece of hyphal growth was taken for sporulation and was confirmed as *Saprolegnia* by microscopic observation, as described earlier. In addition, a piece of hyphal growth was excised and transferred into a 2 mL tube, which was kept at −20 °C until DNA extraction and qPCR, as described previously, to confirm *S. parasitica* infection in the examined fish.

### 2.5. Statistical Analysis

To evaluate the analytical specificity and sensitivity, the threshold in qPCR was determined automatically, as well as, Ct values, standard curve plotting, coefficient of determination (*R*^2^) and efficiency (E = 10^−1/slope^) of each PCR run were analysed using QuantStudio Design & Analysis software (ThermoFisher Scientific, Life Technologies, Carlsbad, CA, USA). The LOD of the qPCR assay was determined as the amount of *S. parasitica* DNA, for which over 95% of positive samples were detected [22]. Variation in qPCR assay results between replicate samples was evaluated from the Ct mean and ±SD of the replicates.

The correlation between the quantity of *S. parasitica* DNA measured by qPCR (ng/L) and the *Saprolegnia* sp. spore number (spores/L) measured with the MWP method was assessed using Pearson’s correlation coefficient with 95% confidence interval (CI) using IBM SPSS Statistic software (version 1.0.0.1447). The respective repeatability was estimated using normalized values and interclass-correlation coefficient (ICC) [23]. Water sample result from tank 1a was excluded from these analyses, as the amount of *Saprolegnia* in the water sample was above the detection limit of the MWP method.

## 3. Results

### 3.1. qPCR Validation

**Figure 1:**
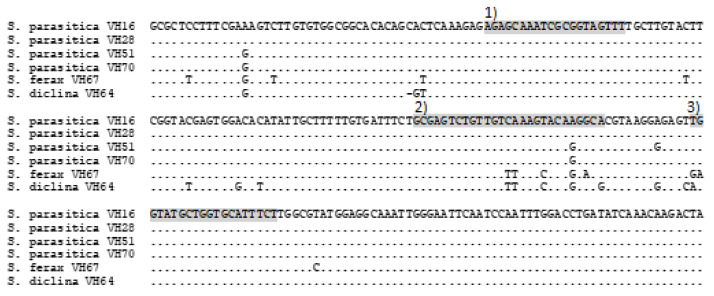
Alignment of the internal transcribed spacer (ITS) region and 28S rRNA gene sequences of oomycetes isolated from salmonid fish, fry and eggs. Shaded in grey are (1) *Saprolegnia parasitica* primer-F, (2) *S. parasitica* probe-R and (3) *S. parasitica* primer-R [11] used in this study for qPCR validation.

When The qPCR primers and probe [11] were compared in silico, the BLAST searches were identical with *S. parasitica* ITS sequences. Some of the identically aligning sequences were named as *Saprolegnia* sp., *S. hypogyna* or *S. salmonis* in GenBank. Currently, *S. hypogyna* and *S. salmonis* are taxonomically designated as *S. parasitica* species according to the study of molecular operational taxonomic units (MOTU) [7]. In addition, the primers and probe were complimentary for all the *S. parasitica* ITS sequences isolated and identified from Finnish fish farms, except for a one-base difference in the probe sequence compared to *S. parasitica* strains formerly known as *S. hypogyna* (e.g., *S. parasitica* strains VH70 and VH51) (Figure 1). The primers and probe were not complementary to other ITS sequences of oomycetes isolated from Finnish fish farms, and the most closely aligning oomycete species sequences were *S. ferax* (7 bp difference) and *S. diclina* (6 bp difference) (Figure 1).

### 3.2. Analytical Specificity and Sensitivity

The qPCR amplified well for all the pure culture samples of *S. parasitica*, including strains VH70 and VH51, which had a difference of one base in the attachment site of the probe used (Figure 1), while none of the other species of oomycete isolates amplified (Table 1).

The results from the sensitivity analysis using 10-fold-diluted DNA isolated from *S. parasitica* VH28 mycelium can be seen in Table 2. The qPCR assays were considered to be optimal, as the standard curve for each qPCR had a coefficient of determination *R*^2^ ≥ 0.98 and efficiency (E) 95–103%. The lowest amount the qPCR detected in each replicate (100%) was 1.8 fg of *S. parasitica* DNA, regardless of whether IAC was present (Table 2). However, the standard deviation of the mean Ct values for the 1.8 fg DNA template was over 1 and there was more variation between qPCR runs than with a higher amount (1.8 ng–18 fg) of DNA, suggesting that quantitative measures of DNA over 18 fg are more accurate than smaller amounts of DNA template. Based on these results, the LOD of the *S. parasitica* qPCR assay was determined as 1.8 fg. Although the LOD was same for the qPCR assays with and without IAC, the Ct values and standard deviations were relatively higher in qPCRs using IAC (Table 2).

### 3.3. Comparison of qPCR and Microbiological Methods for the Detection of S. parasitica

A *Saprolegnia* infection was detected in a moribund fish in tank 4, the *S. parasitica* + injection treatment (Table 3). The fish was killed with an overdose of benzocaine on day 12 and sampled. Whitish lesions on the skin and erosion of the fins were observed. *S. parasitica* was confirmed from fish mucus samples with agar cultivation and with a direct qPCR assay (Table 3). None of the other fish in the same tank or any other tank in the experiment displayed disease signs, and neither were pathogens isolated. Furthermore, no *S. parasitica* was detected from any other fish mucus samples with the direct qPCR assay (N = 59 fish) or following cultivation of mucus on PG-1 plates (N = 92 fish) (Table 3).

All the water samples from experimental fish tanks 1, 2, 3 and 4 with added *S. parasitica* spores that were taken on four different days were positive in qPCR (16 samples, 100% positive for *S. parasitica*) (Table 3). The qPCR assay detected the oomycete as late as 15 days after the addition of *S. parasitica* to tank water (Table 3). The quantification of *S. parasitica* with the qPCR assay revealed that the amount of *S. parasitica* DNA decreased in tanks 1–4 over time (Table 3). PG-1 agar plates inoculated with water samples were *S. parasitica* positive on day 1 in the *S. parasitica* treatment tanks 1 and 2, while on days 4, 7 and 15, *S. parasitica* hyphal growth was not detected on any of the plates (Table 3). In *S. parasitica* + injection treatment tank 3, hyphal growth was only observed for the water sample taken on day 15, while in tank 4 (in which saprolegniosis was detected on one fish), agar plates grew hyphae on days 1, 4 and 15. Of the 16 water samples taken from the *S. parasitica* treatment tanks 1–4, 37.5% were positive in agar cultivation. Water samples from *S. parasitica* treatment tanks 1–2 incubated in PG-1 broth yielded positive results on days 1 and 4 in *S. parasitica*-treated tank 1, while in another *S. parasitica*-treated replicate tank 2, *S. parasitica* hyphal growth was not detected on any of the sampling days in PG-1 broth (Table 3). Water samples from both *S. parasitica* + injection treatment tanks 3 and 4 displayed hyphal growth in broth for the day 1 sample, and tank 4 also had growth on day 3. Thus, out of the total 16 water samples taken from *S. parasitica* tanks 1–4, 31.5% were *S. parasitica* positive in broth cultivation. Incubation of hemp seeds in tank water occasionally resulted in the growth of hyphae in both control water samples and those from tanks with added *S. parasitica*. However, as none of the hyphae were recognised as *Saprolegnia* sp. in the morphological examination, positive samples were not found with the hemp seed method (Table 3), and of the 16 water samples from *S. parasitica* tanks 1–4, 0% were *S. parasitica* positive. All control tanks 5 and 6 water samples were negative in inoculation on PG-1 agar and in PG-1 broth and using the hemp seed method (eight water samples, 100% negative for *S. parasitica* with the microbiological detection methods) (Table 3). The qPCR assay yielded a low positive result in one technical replicate well for one control tank water sample in sampling on day 15. Because all the other replicate analyses from this water sample were negative, this potentially false result was omitted from the analysis, and the seven water samples analysed with qPCR were 100% negative for *S. parasitica*.

### 3.4. S. parasitica in Aquaculture Environments

In two of the fish farms from which fish and water were sampled, saprolegniosis was visually observed on fish. Obviously, these tanks had the high amounts of *S. parasitica,* as was confirmed from the water samples (tanks 1a, 1b, 4a and 4b) with the qPCR assay and the MWP method (Table 4). *S. parasitica* was also detected from the mucus of all the sampled fish using the qPCR assay and microbiological cultivation (Table 4). On the same farms, samples were also taken from tanks in which saprolegniosis was not visually detected (1c and 4c). The amount of *Saprolegnia* in the water samples was lower in these tanks compared to those with diseased fish, and *Saprolegnia* was not detected in fish mucus samples from tank 1c, while in tank 4c, two fish mucus samples were positive in qPCR. However, *Saprolegnia* was not isolated from fish mucus in tank 4c using cultivation on PG-1 agar (Table 4). Farm 3 had no history of *Saprolegnia* outbreaks and *Saprolegnia* was not detected on fish or in water samples, while only very small amounts were detected in the qPCR assay from tanks 3a and 3b (Table 4). Fish farm 2, which used aRAS system, had the highest water temperature and cultivated 0-year-old rainbow trout. *Saprolegnia* was present in the water of all tanks, but saprolegniosis was not visually detected on the farmed fish (Table 4). Neither was *S. parasitica* detected from fish mucus samples by qPCR, but in tank 2b, *Saprolegnia* was isolated from the gill of one fish using PG-1 agar cultivation (Table 4). A small amount of *S. parasitica* was detected in the water sample from Lake Kallavesi using both the qPCR and MWP methods. The standard deviation from the mean for the three filtrates from tank water samples was high when the amount of *S. parasitica* DNA was low in the sample, suggesting that the quantification of very low amounts of *S. parasitica* DNA is somewhat uncertain (Table 4).

**Figure 2:**
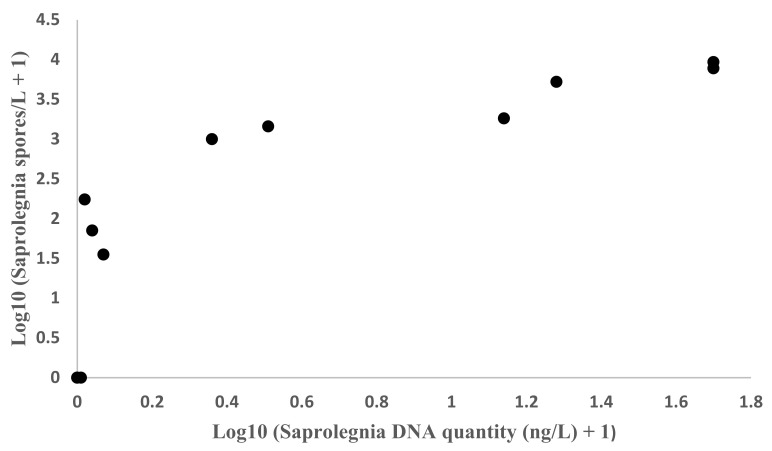
Correlation between the quantity of *S. parasitica* DNA (ng/L) determined with qPCR and the quantity of *Saprolegnia* sp. spores (spores/L) determined using MWP (values for tank 1a excluded as the MWP result could not be determined from the tank water sample, n = 12), Pearson’s correlation coefficient 0.82, *p* = 0.001.

The amount of *Saprolegnia* correlated significantly (Pearson’s correlation coefficient 0.82, N = 12, *p* = 0.001) between qPCR and MWP methods in 12 water samples taken from aquaculture environments and from a lake (Figure 2). The respective repeatability estimate, ICC, between the methods was 0.698 (*p* = 0.003).

## 4. Discussion

We found the qPCR assay developed by Rocchi et al. [11] to perform very well in the detection of *S. parasitica* from both water and fish samples. Thus, the method can be considered validated for practical use in *S. parasitica* eDNA surveillance in water samples and in the diagnostics of the disease in fish and salmonid aquaculture environments. PCR assays recognizing various pathogenic microorganisms from fish are common and important in disease diagnostics, and the use of qPCR assays for pathogen eDNA surveillance, especially in aquatic environments, is rapidly increasing [12,15]. While *S. parasitica* is currently considered one of the major problems, especially in salmonid aquaculture [1,2,3,5], few qPCR assays to detect *S. parasitica* from water samples have been published [11,24]. Furthermore, to our knowledge, the diagnostic recognition of *S. parasitica* from fish with PCR has not been published before this study. However, digital droplet PCR (ddPCR) assay to quantify *S. parasitica* from water and fish samples was recently published [25] and further comparison in vivo of these methods would be interesting.

In silico, specificity analyses of *S. parasitica* sequences can be misleading if they are only interpreted with GenBank data, as misassigned and old species names in the *Saprolegnia* genus exist [7]. Furthermore, GenBank data are always incomplete when compared to the natural environment and the discovery of new species can often lead to the re-evaluation of diagnostic methods [26]. Indeed, we aimed to validate this qPCR assay specifically for the recognition of *S. parasitica* in salmonids in aquaculture. We therefore used ITS sequences, in addition to GenBank sequences, from oomycete cultures collected from fish farms to generate reference sequences of oomycetes from salmonid fish [1] and additionally compared the alignment of primers and probe for them. This approach is also recommended in eDNA method validation, when considering the assay to a new geographic location [16]. Interestingly, in a salmonid oomycete sequence database collected from Finnish fish farms [1], we found *S. parasitica* strains with a one base pair difference in the probe alignment, which could not be observed in GenBank alignments and was not detected in an earlier study [11]. However, in in vitro analytical specificity testing, the qPCR assay detected these strains as well as all the other *S. parasitica* strains tested, so false negative results are unlikely, regardless of this mismatch of one base pair. Furthermore, no cross-reactivity was detected with any of the other *Saprolegnia* species, such as *S. ferax, S. australis, S. diclina, S. torulosa, Saprolegnia* sp. or oomycetes (*Leptolegnia* sp. and *Aphanomyces* spp.) tested in vitro. Mismatches in qPCR assays are quite common because of variation and mutations in genomes. Tuffs and Oidtmann [27] found no impact on qPCR assay sensitivities with sequence difference of one base in the primer and *Aphanomyces astaci* DNA. However, a mismatch in the probe can affect amplification efficiency, so more research should be conducted to evaluate the significance of mismatch in the quantification results for *S. parasitica* strains that have this difference of one base pair. Pavic et al. [25] reported cross reactivity with *S. parasitica* ddPCR assay for *Saprolegnia* sp. 1 strain [7], it seems that this strain has only one base pair difference in reverse-primer used here, so further studies of the specificity with *Saprolegnia* sp. 1 strain in vivo would be useful.

The analytical sensitivity in detecting and quantifying *S. parasitica* with the qPCR assay in this study was similar to the results of Rocchi et al. [11] who determined the quantification limit of the assay as 0.5 fg of *S. parasitica* DNA/µL, when they used 5 µL of DNA template in the PCR reaction. The small difference in comparison to our study could be due different approaches in the assay, such as a different quencher at probe, PCR reaction mix and PCR cycler. It seems that the *S. parasitica* qPCR assay used here has a lower detection limit than that reported for qPCR assays based on amplification of the *A. astaci* ITS region, which have ranged from 50 fg to 160 fg [27,28] or ddPCR assay of *S. parasitica*, which was determined as 14 fg gDNA [25]. The genome of *A. astaci* is larger than that of *S. parasitica,* and multiple ITS regions are present in different species of oomycetes, varying in number even within the species [29], so comparison between different oomycete species and strains is difficult as such.

Comparison of the qPCR assay of water samples with other *S. parasitica* detection methods e.g., microbiological cultivation techniques, demonstrated that the qPCR assay was distinctly the most sensitive method to detect *S. parasitica* from water samples. The amount of *S. parasitica* in water decreased over time, and cultivation methods were typically most suitable to detect *S. parasitica* in the days immediately after the addition of *S. parasitica* to the water. One of the obstacles in culture-based detection methods is the limited sample size used, while in sampling for qPCR, several litres of sample can be filtered, which makes the detection of the pathogen in environmental samples more plausible. However, when using large amounts of water, the accumulation of inhibitors in the sample is also more likely. Indeed, PCR inhibition in environmental samples is one of the most common obstacles to eDNA analysis [14,16]. A feasible method to detect possible inhibition in PCR reactions is to use anIAC, such as the pUC 18 plasmid [18] we included. In our study, use of IAC did not change the LOD of the qPCR assay. However, a downward shift in Ct values was observed in the qPCR assay using IAC. This demonstrates the importance of complete documentation of the qPCR validation process so that such changes can be noticed in the practical use of the assay. PCR inhibition was only detected in a few samples in this study, and these were all water samples. In particular, water samples taken from the lake displayed inhibition and needed to be diluted to achieve qPCR results. It could be that the typically present filtration of upcoming water in aquaculture systems removes some large inhibition resources, for example solids, and as such explains the rarer inhibition in water samples taken from aquaculture environments compared to lake samples. In addition, only 15 mL (as three replicates) of each water sample was filtered in this study, so the amount of potential inhibitory substances was much smaller than when using larger amounts of water to filter, as is most commonly the case in eDNA samples taken from natural waters [14].

In this study, we did not manage to isolate *S. parasitica* from hemp seeds incubated in tank water from the fish experiment. Rocchi et al. [11] used hemp seeds as bait in river water and with cultivation methods also failed to detect *Saprolegnia* growing on the hemp seeds. However, they detected *S. parasitica* from baits when using them as samples in qPCR. It could be that in this study other fungi/oomycetes obstructed the cultivation of *S. parasitica* on hemp seeds. Hemp seeds have been efficiently used to isolate different *Saprolegnia* spp. from environmental water samples [5,30]; however, the selectivity of hemp seeds could be affected by several factors [20]. Furthermore, *Saprolegnia* spp. is isolated from hemp seed more likely when the seeds are incubated in water as long as for 10 days [5,30]. One of the disadvantages of qPCR in pathogen detection is that it does not distinguish between viable and nonviable pathogens, while the cultivation method only detects pathogens that are viable. In this study, when cultivation results with the MWP yielded 0 spores/L of *Saprolegnia* in water sample taken from fish farms, qPCR results were also 0 or 0.001 ng/L of *S. parasitica* DNA, indicating that no or very little nonviable spores were present in the tank water when samples were taken from the surface water of the fish tanks. Furthermore, the qPCR and MWP results correlated highly when *Saprolegnia* was quantified from fish tank water samples, which suggests that nonviable spores are not big confounding variable when estimating amount of *S. parasitica* in fish tanks in flowthrough fish farms. Asexual free-swimming *Saprolegnia* zoospores are produced in high quantities in fish affected by saprolegniosis and are suspected to be most infective to fish [9,31], these are life-stages of *Saprolegnia* that are also most likely present in surface water of fish tanks. Thoen et al. [20] noted that the viability of spores might be one factor affecting the variability in *Saprolegnia* quantification from water under laboratory conditions. However, they also concluded that a confounding factor affecting *Saprolegnia* quantification was the aggregation and uneven distribution of spores in water samples [20]. This was also seen in our study, with generally high standard deviations from the mean for the three MWP cultivations and three filterings from the same water sample taken from fish tanks, even though the samples were always vigorously shaken before taking aliquot samples for analysis. These findings underline the conclusion that quantitative results from MWP and qPCR methods are always estimations of the actual number of *S. parasitica* in water.

Saprolegniosis infection was confirmed only in one fish at the exposure experiment in treatment with *S. parasitica* + injection. The infection was confirmed by qPCR assay and agar cultivation of fish mucus, which were positive for *S. parasitica,* while *S. parasitica* was not isolated from any of the other studied fish mucus samples in the exposure experiment. Successful in vivo *Saprolegnia* infections are usually accompanied by stressful treatment such as netting [32,33], it could be that sham injection and other conditions in the exposure experiment were not stressful enough for fish, e.g., not favourable for *S. parasitica* to cause saprolegniosis in most of the fish. Furthermore, there is variability in pathogenicity of *S. parasitica* strains used in in vivo analysis [32,33]. Similarly, in vivo experiments, fish mucus samples taken from fish farms were often negative, although *S. parasitica* was detected in water samples from the same tanks. In a study on salmon hatcheries even high numbers of *Saprolegnia* spores in water did not have an effect on hatching success [34] and *Saprolegnia* only attached onto dead eggs [35]. These findings support the consensus that *S. parasitica* needs confounding factors, such as stress, wounds or coinfections, to attach to and infect fish [9]. On the other hand, when *S. parasitica* was detected from fish mucus samples, it was always also detected in high amounts in water samples taken from the same tanks as the fish. This is most likely due to the fact that *S. parasitica* produces large amounts of spores in infected fish [31].

In conclusion, we promote the use of qPCR methods in the monitoring of *S. parasitica* in both fish and the environment. Understanding the sources and dynamics of abundance of *S. parasitica* could help in developing drug-free measures to reduce the prevalent problems caused by this disease agent in aquaculture and in wild salmonids.

## Figures and Tables

**Table 1:** Oomycetes from the oomycete culture collection of the Finnish Food Authority used to test the specificity and sensitivity of the *Saprolegnia parasitica* primers and probe.

Oomycete	Strain	Host/Source (Watercourse)	GenBank nro	Amplification
*S. parasitica*	VH28	Brown trout/Vuoksi	OP629478	+
*S. parasitica*	VH123 (P13-2/18)	Brown trout/Kemijoki	OP629475	+
*S. parasitica*	VH16	Rainbow trout/Kymijoki	OP629493	+
*S. parasitica*	VH70	Rainbow trout fry/Vuoksi	OP679802	+
*S. parasitica*	VH51	Rainbow trout egg/Vuoksi	OP648678	+
*S. ferax*	VH67	Rainbow trout fry/Vuoksi	OP623594	−
*S. australis*	VH139 (P4-2/18)	Rainbow trout/Sirppujoki	OP642559	−
*S. diclina*	VH144 (P6-2/18)	Rainbow trout egg/Kymijoki	OP618805	−
*S. diclina*	VH48	Rainbow trout egg/Vuoksi	OP618809	−
*S. diclina*	VH64	Rainbow trout/Vuoksi	OP642454	−
*S. diclina*	VH52	Rainbow trout egg/Vuoksi	OP618810	−
*S. torulosa*	VH40	Rainbow trout/Kymijoki	OP672480	−
*Saprolegnia* sp.	VH53	Rainbow trout egg/Vuoksi	OP679805	−
*Saprolegnia* sp.	VH27	Landlocked salmon/Kymijoki	OP648311	−
*Aphanomyces laevis*	6275/07	Signal crayfish/Vuoksi	OP659017	−
*A. repetans*	606/14	Pikeperch/Kymijoki		−
*A. astaci*	Da	Noble crayfish/Sweden	QUTH00000000.1	−
*A. astaci*	Si	Noble crayfish/Sweden	QUTB00000000.1	−

**Table 2:** ITS real-time quantitative PCR (qPCR) assay sensitivity results using DNA from *S. parasitica* mycelium with or without internal amplification control (IAC) (n = 6).

DNA Quantity	No IAC			With IAC		
	C_t_ Mean	Standard Deviation	% Detection	C_t_ Mean	Standard Deviation	% Detection
1.8 ng	14.40	0.65	100	15.81	0.46	100
0.18 ng	17.22	0.22	100	19.33	0.44	100
18 pg	20.56	0.27	100	22.56	0.36	100
1.8 pg	23.95	0.25	100	26.15	0.44	100
180 fg	27.69	0.24	100	29.42	0.49	100
18 fg	31.39	0.27	100	33.15	0.56	100
1.8 fg	34.24	1.03	100	37.07	1.04	100
0. 18 fg	36.11	0.32	50	38.47	0.16	33

**Table 3:** Detection of *S. parasitica* from water and fish samples with qPCR and microbiological methods in an *S. parasitica* exposure experiment, in which 17 fish per tank, in four tanks (1–4), where exposed to *S. parasitica* spores (concentration 3 × 10^4^ spores/L) and two control tanks (5 and 6) received sterile MilliQ water. Exposed fish in the tanks 3 and 4 were additionally sham-injected to mimic vaccination stress.

Treatment	Day	*Saprolegnia parasitica* Detection Method											
		qPCR Water (DNA ng/L)		Agar Water		Broth Water		Hemp Seed Water		PCR Fish ^1^		Agar Fish ^1^	
*S. parasitica*		Tank 1	Tank 2	Tank 1	Tank 2	Tank 1	Tank 2	Tank 1	Tank 2	Tank 1	Tank 2	Tank 1	Tank 2
	1	96.4	169.6	+/− ^2^	+/+	+/+	−/−	−/−	−/−				
	4	5.6	3.2	−/−	−/−	+/−	−/−	−/−	−/−				
	7	1.0	0.3	−/−	−/−	−/−	−/−	−/−	−/−				
	15	1.2	0.6	−/−	−/−	−/−	−/−	−/−	−/−	0/10	0/10	0/14	0/16
*S. parasitica* + injection		Tank 3	Tank 4	Tank 3	Tank 4	Tank 3	Tank 4	Tank 3	Tank 4	Tank 3	Tank 4	Tank 3	Tank 4
	1	113.3	82.3	−/−	+/+	+/+	+/+	−/−	−/−				
	4	3.0	5.4	−/−	+/−	−/−	+/−	−/−	−/−				
	7	1.5	4.2	−/−	−/−	−/−	−/−	−/−	−/−				
	15	0.3	1.1	+/−	+/−	−/−	−/−	−/−	−/−	0/10	1/10	0/15	1/13
Control		Tank 5	Tank 6	Tank 5	Tank 6	Tank 5	Tank 6	Tank 5	Tank 6	Tank 5	Tank 6	Tank 5	Tank 6
	1	0.0	0.0	−/−	−/−	−/−	−/−	−/−	−/−				
	4	0.0	0.0	−/−	−/−	−/−	−/−	−/−	−/−				
	7	0.0	0.0	−/−	−/−	−/−	−/−	−/−	−/−				
	15	0.0	− ^3^	−/−	−/−	−/−	−/−	−/−	−/−	0/10	0/10	0/23	0/12

^1^ No. of *Saprolegnia*-positive fish/no. of studied fish; ^2^ Positive/negative results of *Saprolegnia* growth in duplicate medium; ^3^ Omitted.

**Table 4:** Details of sampling and results from four fish farms, from which fish and water were sampled and examined for the presence of *Saprolegnia*. A lake water sample was included as a reference. Sampling date, water temperature and sampled fish and age in each farm is included. Samples were taken from three tanks in each fish farm (a–c), where fish abundance in each tank was determined. *Saprolegnia* in fish mucus samples were compared by PCR and agar methods. The average and standard deviation (±SD) were calculated for each tank water sample from 3 replicate 96-well plates with microwell plate (MWP) enumeration or from three filtrations withqPCR assay.

Fish Farm	Sampling Date	Water Temperature (°C)	Fish Species	Fish Age	Tank	Fish Abundance in Tank kg/m^3^	*Saprolegnia* in Fish Confirmed with Skin Mucus PCR ^2^	*Saprolegnia* in Fish Isolated on Agar ^2^	MWP *Saprolegnia* Spores/L (n = 3)	qPCR *Saprolegnia* DNA ng/L (n = 3)
**1**	6 May 2020	5.8	Brown trout	2	1a	6	5/5	5/5	>10,000	1450.9 ± 120.6
					1b	ND ^1^	5/5	5/5	9410 ± 159	49.2 ± 16.5
					1c	0.5	0/5	0/5	35 ± 60	0.2 ± 0.2
**2**	18 May 2020	16.3	Rainbow trout	0	2a	32	0/5	0/5	1007 ± 262	1.3 ± 0.4
					2b	36	0/5	1/5	1806 ± 159	12.7 ± 3.0
					2c	18	0/5	0/5	1458 ± 853	2.2 ± 0.3
**3**	25 May 2020	12.7	Brown trout	1	3a	4.4	0/5	0/5	0 ± 0	0.01 ± 0.02
					3b	1.2	0/5	0/5	0 ± 0	0.01 ± 0.01
					3c	7.4	0/5	0/5	0 ± 0	0 ± 0
**4**	27 May 2020	14	Landlocked salmon	1	4a	4.3	5/5	5/5	5278 ± 481	17.95 ± 15.5
					4b	5.1	5/5	5/5	7813 ± 276	49.35 ± 16.5
					4c	0.5	2/5	0/5	174 ± 159	0.06 ± 0.05
**Lake**	1 June 2020	15.8							69 ± 60	0.1 ± 0.07

^1^ ND = Not determined; ^2^ No. of *Saprolegnia*-positive fish/no. of studied fish.

## Data Availability

All data is available in this paper, through GenBank with accession numbers and upon request.
